# Evaluating the Bioactivity of a Novel Antimicrobial and Anticancer Peptide, Dermaseptin-PS4(Der-PS4), from the Skin Secretion of *Phyllomedusa sauvagii*

**DOI:** 10.3390/molecules24162974

**Published:** 2019-08-16

**Authors:** Dong Chen, Xiaowei Zhou, Xi Chen, Linyuan Huang, Xinping Xi, Chengbang Ma, Mei Zhou, Lei Wang, Tianbao Chen

**Affiliations:** 1 Natural Drug Discovery Group, School of Pharmacy, Queen’s University, Belfast BT9 7BL, UK; 2 Department of Nutrition, Henry Fok School of Food Science and Engineering, Shaoguan University, Shaoguan 512005, China; 3 School of Life Sciences and Technology, China Pharmaceutical University, Nanjing 211198, China

**Keywords:** dermaseptin, molecular cloning, antimicrobial peptide, anticancer peptide, frog skin secretion

## Abstract

Dermaseptins belonging to a large family of cationic membrane-disruption antimicrobial peptides display extensive antibacterial and antiproliferative activities depending on a coil-to-helix transition and the specific structural parameters. Herein, a novel dermaseptin peptide named Der-PS4 was discovered from the skin secretion of the waxy monkey tree frog, *Phyllomedusa sauvagii*. The complementary DNA (cDNA)-encoding precursor was obtained relying on “shotgun” cloning, and afterwards, a mature peptide amino acid sequence was identified by reverse-phase high performance liquid chromatography (RP-HPLC) and MS/MS. Specimens were chemically synthesized and applied for further functional studies. Structural analysis demonstrated a higher α-helical content in the membrane-mimetic environment compared with that in the ammonium acetate/water circumstance. Der-PS4 displayed a broad spectrum of antimicrobial activities against tested pathogenic microorganisms, however, exhibiting slight membrane-damaging effectiveness towards horse red blood cells. Coincident with the inhibitory activities on pathogens, Der-PS4 also showed considerable biofilm eradicating impact. Also, Der-PS4 penetrated cell membrane in a relative short period under each minimum bactericidal concentration. In addition, Der-PS4 possessed antiproliferative capacity against five cancer cell lines, while presenting slight suppressing effect on human microvascular endothelial, HMEC-1. These findings provide a promising insight for the discovery and development of novel drugs from a natural source.

## 1. Introduction

The host-defense peptides isolated from anura (frog and toad) skins have promising therapeutic applications and have attracted much attention owing to their multiple biological activities. This abundant natural product resource shows multiple physiological and pathological functions in the form of bactericidal, antiproliferative, immunoregulatory, and antidiabetic properties [1]. Actually, these kinds of host-defense peptides not only exist in frog species but also can be discovered from human bodies, microorganisms, and plants [2]. The existence of frog skins and secretions are always considered as the first barrier for these creatures to defend against predators or protecting them from invading microbes in the environment [3]. Recent research suggests that symbiotic bacteria on skins play a more important role for survival, while host-defense peptides may act as innate immunomodulators to assist symbiotic bacteria to defend against pathogens in some species [1,4,5]. The peptides isolated from frog skins vary in size from a minimum of eight residues to a maximum of forty-eight residues. Some mutual physicochemical properties, including a cationic charge, amphipathicity, and α-helical structures are ubiquitous. However, structural studies suggest that more α-helical content would be observed in the membrane-mimetic environment which may provide support for the combination with cellular phospholipid membrane [6]. At the genetic level, highly conserved signal peptide sequences with a particular amino acid replacement and consensus motif in one peptide family from widely distributed frog species may offer some hint at evolutionary processes [7,8].

Dermaseptins, in a narrow sense, refers to a family of antimicrobial or tumouricidal peptides with highly conserved signal sequences and several consensus motifs in mature sequences isolated from the skin or skin secretions of Hylidae and Ranidae frogs [9]. The first dermaseptin, a 34-mer polypeptide named Dermaseptin-S1, was identified from the dried skin of the waxy monkey tree frog, *Phyllomedusa sauvagii,* by Mor and co-workers in 1991 [10]. Followed by functional study, Dermaseptin-S1 exhibited fatal activity against filamentous fungi, which acted as the first gene-coding eukaryotic anti-fungal peptide [9]. On the whole, the prototype members of this family have typical coil-to-helix structures with cationic charge which could facilitate the combination with the anionic membranes, causing penetration and disruption of microorganisms [9,11]. However, the low toxicity to mammalian cells of dermaseptins provide a new concept of membrane selectivity of Dermaseptins. Few years after the discovery of Dermaeptin-S1, another novel peptide named Dermaseptin B2 was identified from the skin of *Phyllomedusa bicolor*, which was an adenoregulin interacting with the adenosine receptor. Owing to the different functions, these two peptides were considered unrelated until the prepropeptides were cloned, which revealed the connection between these two polypeptides according to the highly conserved sequences [12,13,14]. From then on, identifying dermaseptins based on molecular cloning, common regions were constructed, and the dermaseptin family increased progressively. For instance, Dermaseptin-PH and Dermaseptin-PS3 (Der-PS3) were identified from the skin secretion of *Pitheocopus* (*Phyllomedusa*) *hypochondrialis* and *Phyllomedusa sauvagii*, respectively [15,16].

In this present study, a novel antimicrobial and anticancer peptide named Dermaseptin-PS4 (Der-PS4) was isolated from the skin secretion of the waxy monkey tree frog, *Phyllomedusa sauvagii*. The cDNA encoding biosynthetic precursor was identified by “shotgun” cloning and the amino acid sequence was confirmed by RP-HPLC and mass spectrum (MS/MS) fragmentation sequencing via analyzing the crude skin secretions. Der-PS4 applied in functional studies was chemically synthesized by solid-phase peptide synthesis (SPPS) technique. Subsequently, synthetic Der-PS4 was subjected to antimicrobial susceptibility tests by utilizing Gram-positive and Gram-negative bacteria, and fungus. An antiproliferative assay was performed to evaluate the anticancer activities of Der-PS4 against five human cancer cell lines. A haemolysis test and human dermal microvascular endothelium cells were performed to evaluate the cytotoxicity of Der-PS4 against eukaryotic cells.

## 2. Results

### 2.1. Molecular Cloning and Structural Characterisation of Der-PS4 from a Skin Secretion-Derived cDNA Library of Phyllomedusa sauvagii

**Figure 1:**
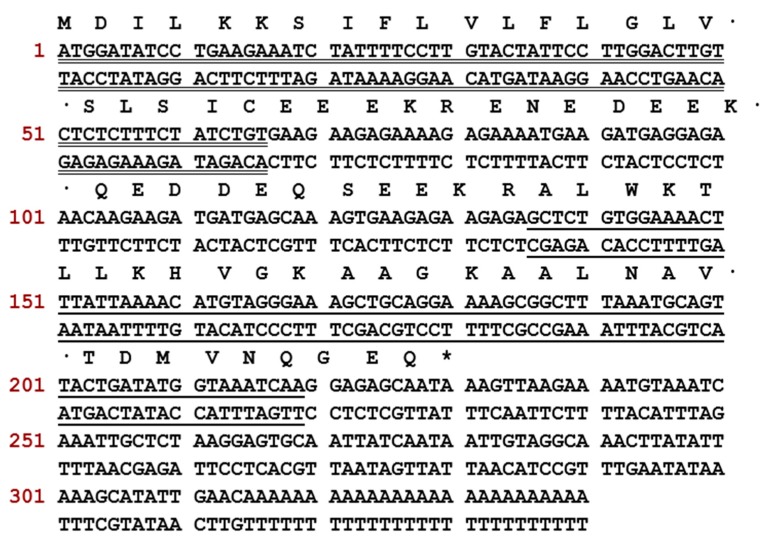
Nucleotide sequence and related open-reading frame of Der-PS4 from the skin secretion of *Phyllomedusa sauvagii*. The putative signal peptide is double-underlined, the mature peptide is single-underlined and the stop codon is marked by an asterisk.

A cDNA encoding biosynthetic precursor of Der-PS4 was identified from the cDNA library with the assistance of a specific degenerate primer designed based on the highly conserved sequence of dermaseptins. The translated open reading frame of Der-PS4 consisted of 340 base pairs encoding 76 amino acids including a highly conserved hydrophobic signal peptide domain of 22 amino acid residues, an acidic spacer containing 16 amino acids and a representative Lys-Arg processing site followed by the predicted mature peptide Der-PS4 comprising 28 amino acids (Figure 1). A consensus motif -GEQ after the mature peptide region estimated a C-terminal post-translational amide modification by comparing the sequences published. The nucleotide biosynthetic precursor of Der-PS4 was submitted to the GenBank Database (https://www.ncbi.nlm.nih.gov/genbank/) under the accession number MK256942.

**Figure 2:**
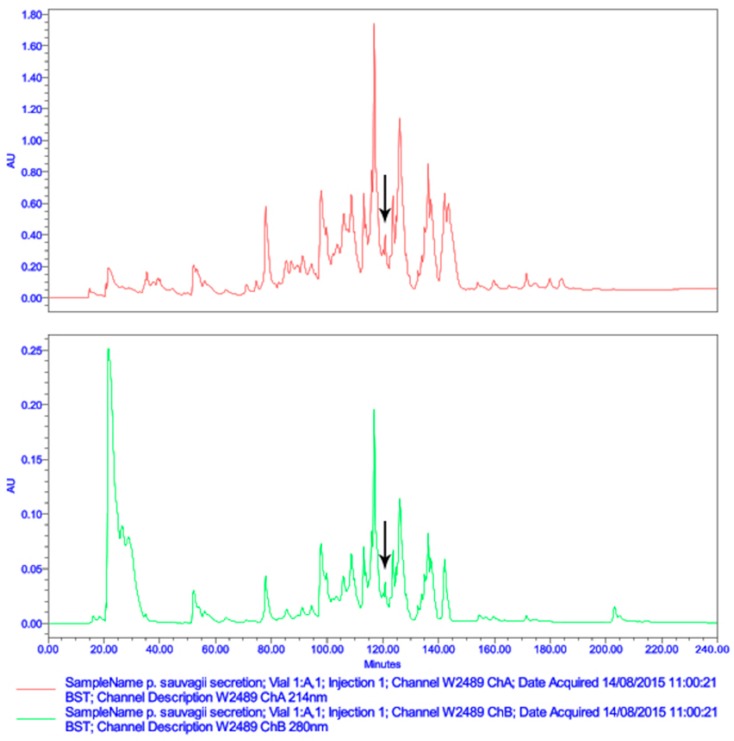
Reverse-phase HPLC chromatogram of lyophilized skin secretion of *Phyllomedusa sauvagii*. The arrow indicates the retention time/elution position of the fraction containing a mass corresponding to the predicted mature peptide. The absorbance was measured at λ = 214 nm and λ = 280 nm. The X-axis indicates the retention time in minutes and the Y-axis indicates the absorbance in arbitrary units.

**Figure 3:**
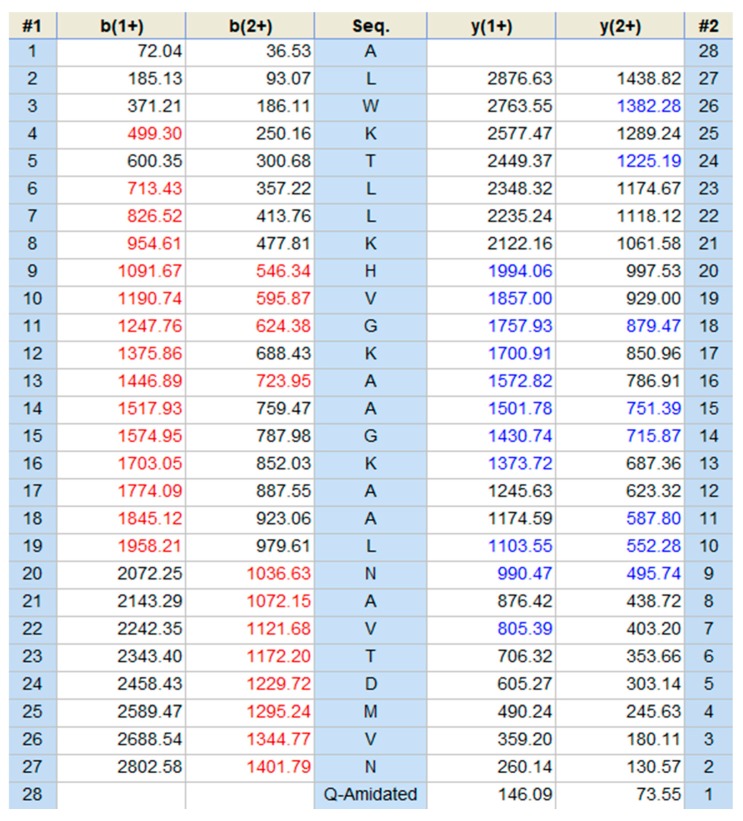
Electrospray ion-trap MS/MS fragmentation data derived from fragment ions corresponding to molecular mass to Der-PS4. Predicted singly and doubly charged *b*-ions and *y*-ions are in black typeface. Ions detected by MS/MS fragmentation are indicated in red and blue.

The expected mature peptide, Der-PS4, was eluted from reverse-phase HPLC by analyzing the lyophilized crude skin secretion of *Phyllomedusa sauvagii* (Figure 2). Fractions collected every minute were analyzed by MALDI-TOF-MS and the one with a mass corresponding to the mature peptide identified by molecular cloning was indicated by an arrow. The amino acid sequence of Der-PS4 was further verified by analyzing the fraction containing estimated peptide mass through utilizing MS/MS fragmentation sequencing technique and result was shown in Figure 3. The predicted post-translational amidation of Der-PS4 with -GEQ residues at C-terminus of biosynthetic precursor was also confirmed in the result.

### 2.2. Secondary Structure Prediction and Structrual Parameters Analysis of Der-PS4

**Figure 4:**
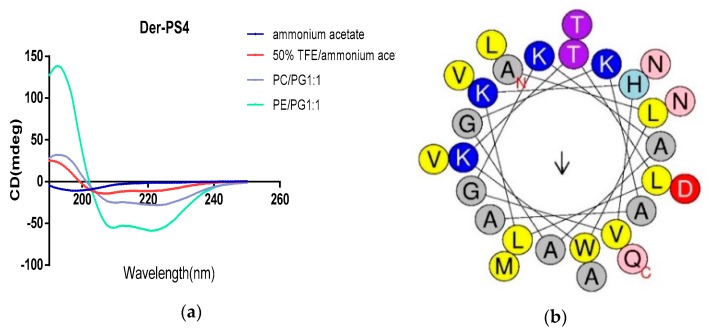
The CD spectra recorded for Der-PS4 in ammonium acetate, 50% TFE/ammonium acetate and membrane mimicking liposomes (**a**). The helical wheel projections of Der-PS4 with an arrow indicated the hydrophobic face (**b**).

The secondary structures of Der-PS4 were determined by circular dichroism spectroscopy in aqueous solution, 50% TFE-water solution and different proportion of DOPE, DOPC and DOPG. (Figure 4a). Der-PS4 was unable to form α-helical structure in ammonium acetate solution with helix value of 4.1. However, in the TFE/ammonium acetate solution and membrane mimicking liposomes, Der-PS4 displayed strong α-helical conformations of 67.6% (TFE/ammonium acetate), 62% (DOPC/DOPG, 1/1) and 77.3% (DOPE/DOPG, 1/1), which indicated Der-PS4 displayed more α-helical content in the membrane-mimetic environment. What’s more, the α-helical wheel plots and physicochemical properties were estimated by Heliquest in Figure 4b and Table 1. The hydrophobicity of Der-PS4 was 0.368 and the hydrophobic moment was 0.358. With four basic amino acids, lysine, and one acidic amino acid, asparticacid, the final net charge was +3. The hydrophobic face of Der-PS4 was predicted by arrow in Figure 4b and consisted with Trp (tryptophan), Ala (alanine), Ala, Leu (leucine), Met (methionine) and Ala. The proportion of polar residues was 46.43% comprising eight uncharged residues and five charged residues.

### 2.3. Antimicrobial Minimal Inhibitory Concentration Assay

**Figure 5:**
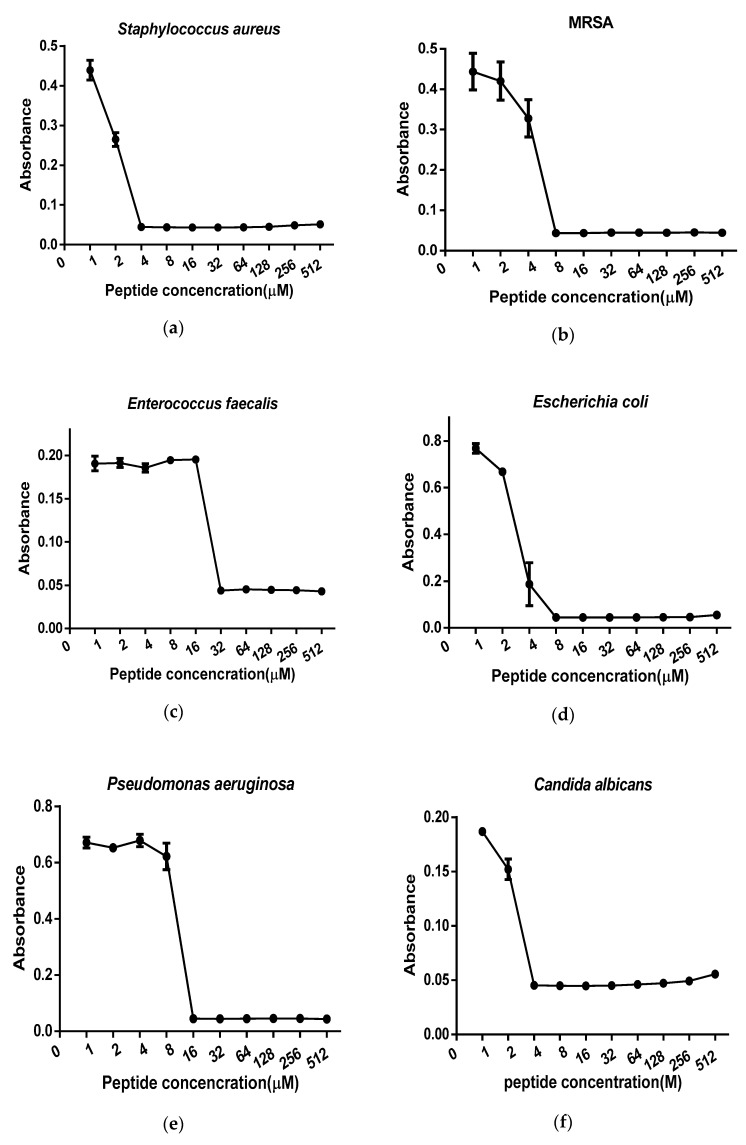
Minimum inhibitory concentrations (MICs) curves obtained following incubation of Der-PS4 with *S. aureus* (**a**), MRSA (**b**), *E. faecalis* (**c**), *E. coli* (**d**), *P. aeruginosa* (**e**) and *C. albicans* (**f**) in a range of concentrations from 1 µM to 512 µM. Data represent means ± SEM (standard error of the mean) of 5 replicates.

**Figure 6:**
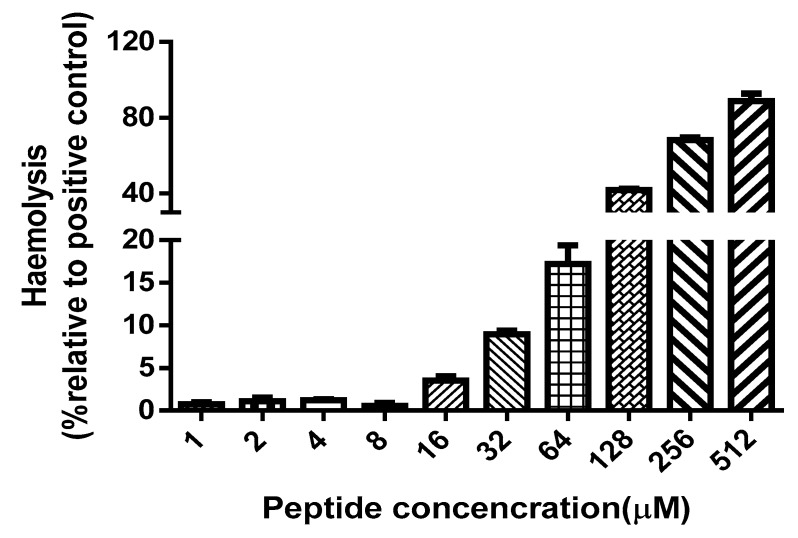
The haemolysis activity of Der-PS4 was tested using horse red blood cells in a range of concentrations from 1 μM to 512 μM. Positive control group was treated with 1% Triton X-100 lysis buffer. Data represent means ± SEM (standard error of the mean) of 5 replicates.

The data derived from minimum inhibitory concentrations (MICs) and minimum bactericidal concentrations (MBCs) of synthetic peptide Der-PS4, melittin, ampicillin and norfloxacin were summarized in Table 2. Der-PS4 was verified to have different antimicrobial activities against Gram-positive bacteria *Staphylococcus aureus* (ATCC 12600), MRSA (ATCC BAA-1720), *Enterococcus faecalis* (ATCC 29212), Gram-negative bacteria *Escherichia coli* (ATCC 11755), *Pseudomonas aeruginosa* (ATCC 27853), and pathogenic fungus *Candida albicans* (ATCC 10231), with MICs ranging from 4 to 32 µM (Figure 5) and MBCs in a range from 8 to 32 µM. For Gram-positive bacteria, Der-PS4 exhibited more effective antimicrobial activity against *S. aureus* and drug-resistant bacterium MRSA with MICs of 4 µM and 8 µM. While, the MIC and MBC of *E. faecalis* were both 32 µM. Although ampicillin showed more efficient antibacterial ability than Der-PS4 on *S. aureus* and *E. faecalis*, it showed no obvious inhibiting effect on MRSA. For Gram-negative bacteria and fungus, Der-PS4 was more efficiency than ampicillin, but weaker than norfloxacin. The MICs and MBCs of melittin were displayed below as a relevant control. Additionally, it was worth noting that although Der-PS4 showed relative 50% haemolyltic activity at around concentration of 128 µM, it displayed slight haemolysis at MICs (Figure 6).

### 2.4. Anti-Biofilm Activity of Der-PS4

Synthetic peptide Der-PS4 exhibited anti-biofilm activity against several microorganisms including Gram-positive bacteria and Gram-negative bacteria (Table 3). The MBIC results of Der-PS4 on *S. aureus*, MRSA and *E. coli* were coincident with the MIC results, with 4, 8, and 8 μM each. However, the MBIC values on *E. faecalis* and *P. aeruginosa* were two times higher than their MIC results, with concentrations of 64 and 32 μM, respectively. For the biofilm eradicating activity, eight times higher values than MBIC values on MRSA and *P. aeruginosa* were obtained. For *E. faecalis* and *E. coli*, the MBEC results were two times and four times higher than MBICs, with 128 μM and 32 μM respectively. However, the MBEC value of *S. aureus* was 64 μM, which was sixteen times higher than its MBIC value.

### 2.5. Bacteria Cell Membrane Permeabilized Activity of Der-PS4

**Figure 7:**
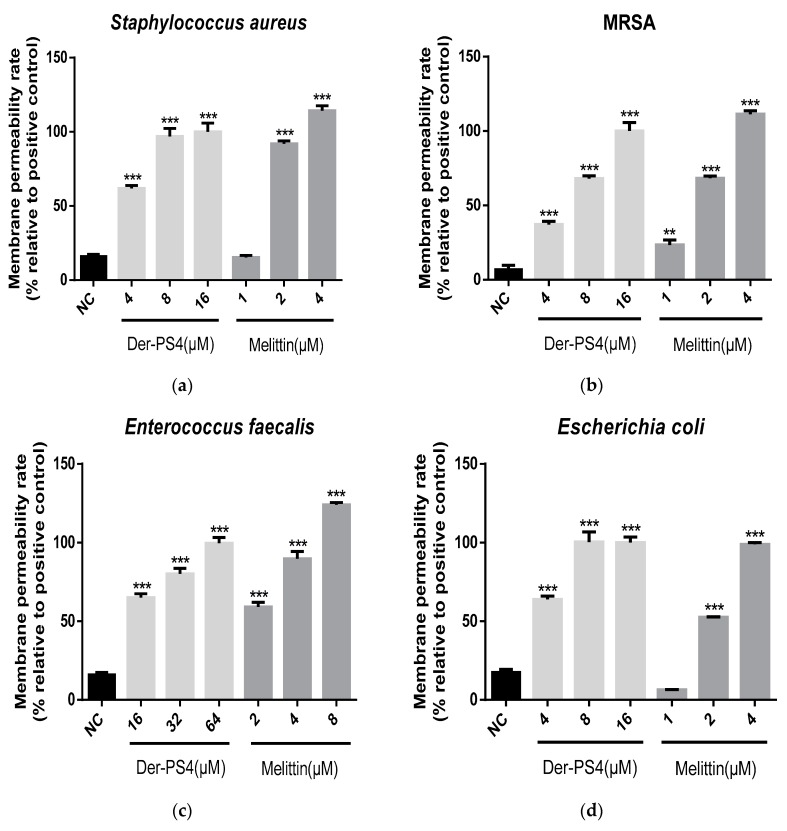

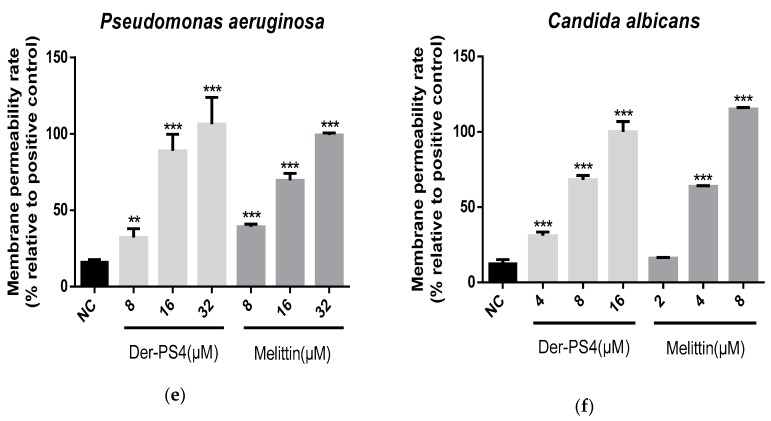
The permeabilization activities of Der-PS4 were evaluated on *S. aureus* (**a**), MRSA (**b**), *E. faecalis* (**c**), E. coli (**d**), *P. aeruginosa* (**e**) and *C. albicans* (**f**). Negative control (NC) was acquired after incubation with 5% tryptic soy broth (TSB) in 0.85% NaCl. Positive control was acquired after incubation with 70% isopropanol. Data represent means ±SEM (standard error of the mean) of five replicates. The levels of significance are: ** *p* < 0.01, *** *p* < 0.001.

The permeabilization activities of Der-PS4 were evaluated in a range of concentrations on bacteria and fungus. Melittin was acted as a relevant control (Figure 7). For Gram-positive bacteria, Der-PS4 displayed stronger permeability on *S. aureus* and MRSA than on *E. faecalis*. At the concentration of 8 µM, cell membrane of *S. aureus* was fully disruptive and that of MRSA was approximately seventy percent permeabilized. However, *E. faecalis* showed no significant permeability rate compared to the negative control under the concentration of 8 µM. One hundred percent of permeability rate reached until 64 µM of Der-PS4 was administrated. For Gram-negative bacteria, the penetration capacity of Der-PS4 was more powerful on *E. coli* than on *P. aeruginosa*, with complete penetration under the concentration of 8 µM on *E. coli* and 32 µM on *P. aeruginosa*. For fungus *C. albicans*, full permeabilization was obtained under the concentration of 16 µM.

**Figure 8:**
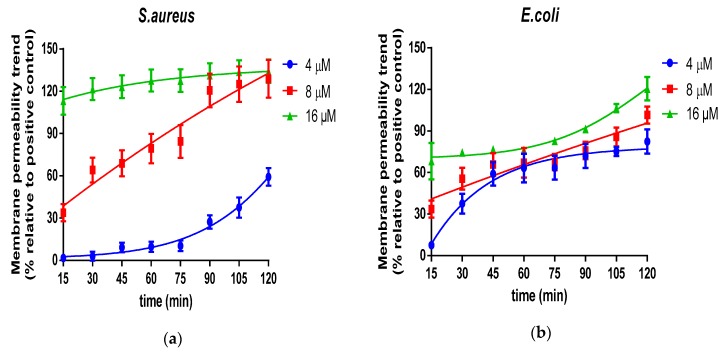

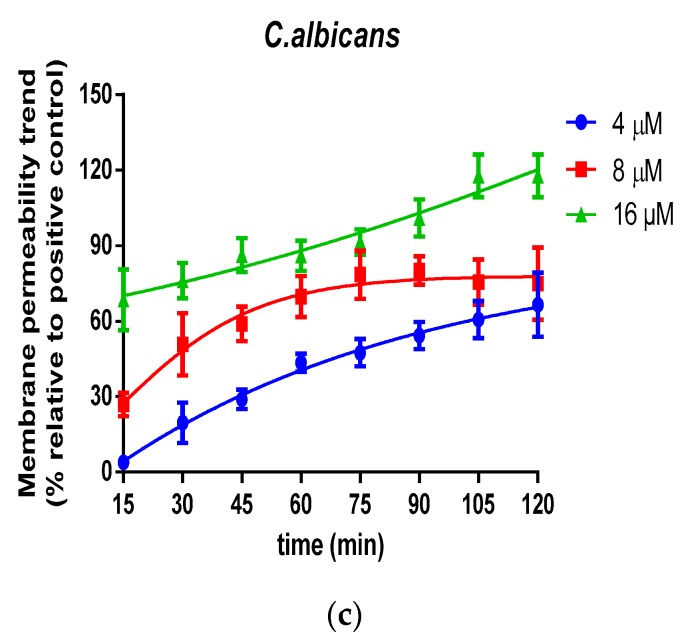
The time-curve of cell-membrane permeabilized activities of Der-PS4 were performed on *S. aureus* (**a**), *E. coli* (**b**) and *C. albicans* (**c**) in a range of concentrations based on the results of end-point on 120 min. The fluorescence was measured every 15 min. Positive control was acquired after incubation with 70% isopropanol. Data represent means ±SEM (stand error of the mean) of five replicates.

What’s more, the real-time dynamic permeable trend was collected on three representative microorganisms, *S. aureus*, *E. coli* and *C. albicans* (Figure 8). Coincident with the end-point results, the efficiently complete permeabilization of Der-PS4 on *S. aureus* and *E. coli* was both 8 µM. However, it’s worth noting that, although Der-PS4 exhibited identical permeable activities on these two microorganisms, the entire penetration of *S. aureus* was accomplished in a relatively short period of time. While, *E. coli* may require a little longer time to reach total penetration. For *C. albicans*, complete permeable trend was achieved at around 90 min after incubation with Der-PS4 and neither 4 µM nor 8 µM of Der-PS4 could thoroughly disrupt the fungal cell membrane.

### 2.6. Scanning Electron Microscopy (SEM) Characterisation

**Figure 9:**
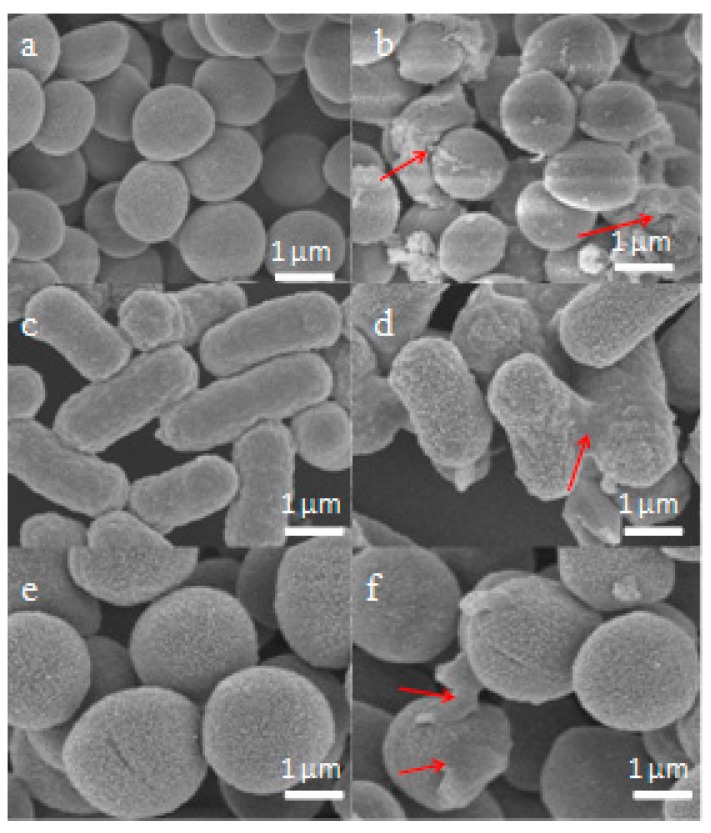
Scanning electron microscopy (SEM) micrographs of *S. aureus*, *E. coli* and *C. albicans* cells treated with peptides at their 1×MICs at 15 min. *S. aureus*: (**a**) control; (**b**) Der-PS4. *E. coli*: (**c**) control; (**d**) Der-PS4. *C. albicans*: (**e**) control; (**f**) Der-PS4. The control group did not contain peptides.

The membrane integrity and morphological changes of cells treated with Der-PS4 were investigated by SEM. As shown in Figure 9a,c,e, it is clearly revealed that *S. aureus*, *E. coli* and *C. albicans* cells display a smooth and distinct surface, however, the membrane of *S. aureus*, *E. coli* and *C. albicans* cells were damaged dramatically in the presence of peptides. We observed that partial *S. aureus*, *E. coli* and *C. albicans* cells exhibited obvious irregularities, shrinkages, blebbing, and a budding surface after treating with the Der-PS4 within 15 min, as in Figure 9b,d,f.

### 2.7. Antiproliferative Activity of Der-PS4

**Figure 10:**
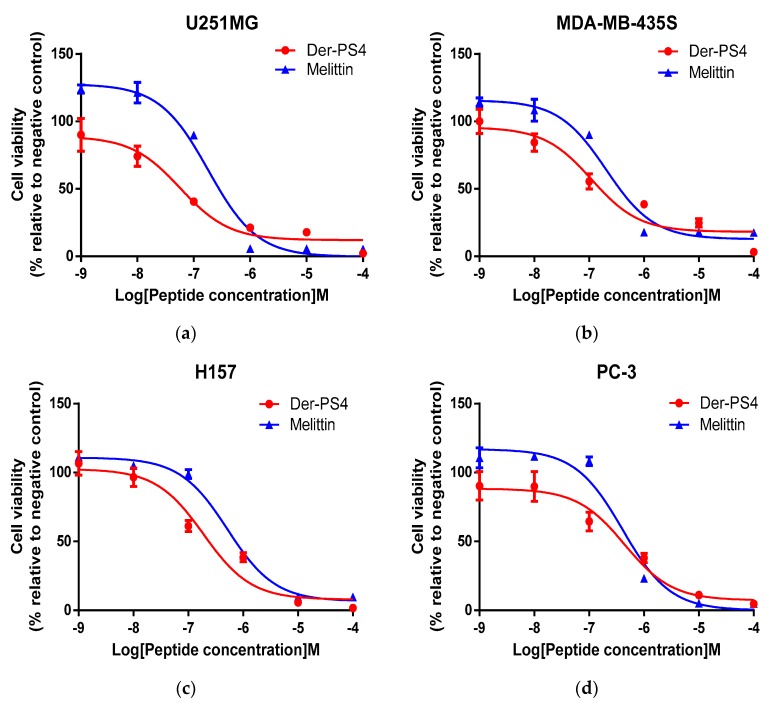

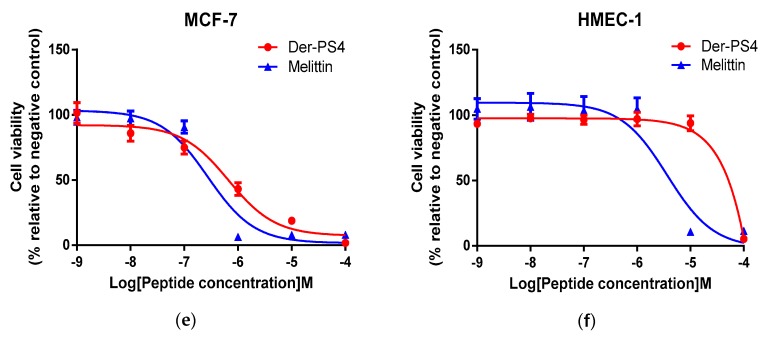
The effect of Der-PS4 on proliferation of human cancer cell lines: U251MG (**a**), MDA-MB-435S (**b**), H157 (**c**), PC-3 (**d**), MCF-7 (**e**) and human dermal microvascular endothelium cell HMEC-1 (**f**) after treated with Der-PS4 for 24 h in a range of concentrations from 10^−9^ to 10^−4^ M. Negative control was acquired after incubation with serum-free culture medium. Data represent means ± SEM (stand error of the mean) of five replicates.

The anticancer activity of Der-PS4 was evaluated on five human cell lines including U251MG, MDA-MB-435S, H157, PC-3, MCF-7, which displayed does-dependent inhibitory activities in a range of concentrations from 10^−9^ to 10^−4^ M (Figure 10). The anticancer activity of melittin is also shown below as a relevant control. Overall, Der-PS4 showed more effective antiproliferative activity than melittin on U251MG, MDA-MB-435S, and H157 cell lines and displayed a comparative effect on PC-3. However, melittin exhibited a stronger inhibitory effect than Der-PS4 on MCF-7. The most effective anti-proliferation activity of Der-PS4 was on the human glioblastoma astrocytoma cell line (U251MG), with an IC_50_ value of 57.66 nM. Der-PS4 exhibited a similar potential against non-small cell lung cancer (H157) and melanoma (MDA-MB-435S), with IC_50_ values of 0.19 and 0.11 µM. For breast cancer (MCF-7) and prostatic cancer (PC-3), the inhibitory activities of Der-PS4 were relatively less effective, with IC_50_ values of 0.67 and 0.44 µM. Additionally, the antiproliferative effect of Der-PS4 was also examined on normal human cells, human dermal microvascular endothelium cells (HMEC-1). Der-PS4 showed slight inhibitory effect (IC_50_ 0.46 M) compared to the potently inhibitory influence on cancer cell lines.

**Figure 11:**
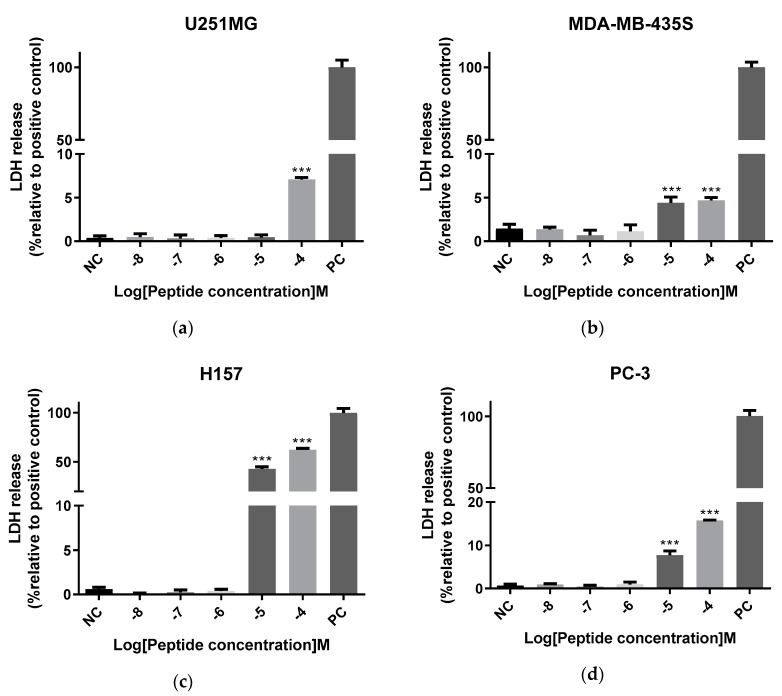

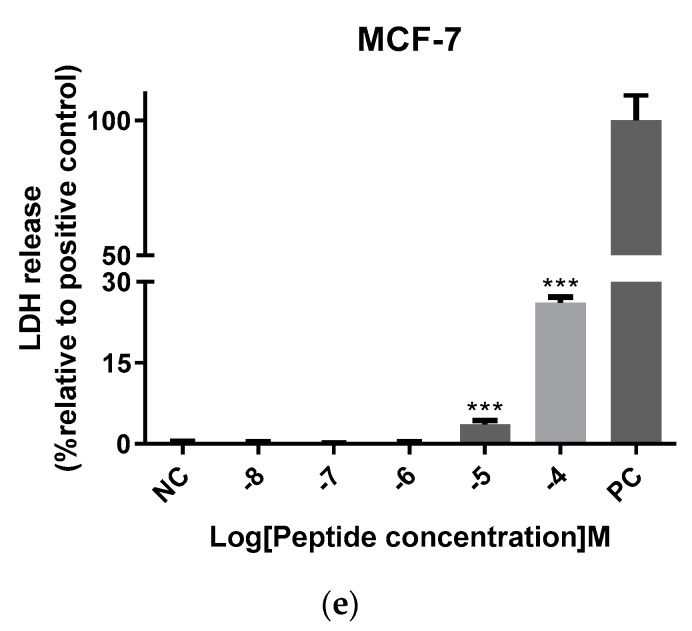
The lactate dehydrogenase (LDH) cytotoxicity results of Der-PS4 on five human cancer cell lines: U251MG (**a**), MDA-MB-435S (**b**), H157 (**c**), PC-3 (**d**) and MCF-7 (**e**). Each cell line was treated with Der-PS4 in a range of concentrations from 10^−8^ to 10^−4^ based on the results of MTT. Negative control was acquired after treated with serum-free culture medium. Positive control was acquired after treatment with lysis buffer supplied from Pierce LDH Cytotoxicity Assay Kit. Data represent means ± SEM (stand error of the mean) of five replicates. The levels of significance are: *** *p* < 0.0001.

The cell membrane integrity of cancer cell lines was tested by a lactate dehydrogenase (LDH) cytotoxicity assay. Based on MTT results, cancer cells were exposed in a range of concentrations from 10^−8^ to 10^−4^ M. According to the results, displayed in Figure 11, MDA-MB-435S, H157 and PC-3 were more sensitive to Der-PS4, with a significant difference at 10^−5^ and 10^−4^ M, while only 10^−4^ M of Der-PS4 could disrupt the cell membranes of U251MG and MCF-7 and induce cell lysis.

## 3. Discussion

Antimicrobial peptides are widespread in most amphibian species. These cationic dermal peptides are structurally diverse and express various levels of antimicrobial activities. Dermaseptins, one family of amphibian antimicrobial peptides, including over 200 analogues known to date are widely distributed in Hylidae frogs, especially in frogs of Phyllomedusinae [9]. In this study, we identified a novel dermaseptin peptide named Der-PS4, using the same degenerate primer as previous reported, from the skin secretions of *Phyllomedusa sauvagii* from which over 50 kinds of bioactive peptides were discovered [17]. Considering Der-PS3 and Der-PS4 are identified in the same frog species (*Phyllomedusa sauvagii*) [16], our results suggested that the signal peptide domain of precursor as well as the processing site of dermaseptins peptides may provide a new approach in the classification of peptides. Although dermaseptins are structurally multifarious, they share a typical conformation containing a tryptophan at the third position, an -AA(A/G)KAA- consensus motif in the middle and an amide post-translational modification at C-terminus [18]. This C-terminal amidation could increase the positive charge of Der-PS4 and avoid degradation in the natural environment [19].

In recent decades, due to the overuse of antibiotics, drug-resistance has become an acute problem in clinical treatment. Pandrug-resistant bacteria such as *Pseudomonas aeruginosa*, extensively drug-resistant bacteria like *Mycobacterium tuberculosis* or multidrug-resistant bacteria such as methicillin-resistant *Staphylococcus aureus* (MRSA), all these resistant pathogens cause serious infections and bring about increasing mortality [20,21,22,23]. Der-PS4 exhibited a broad-spectrum of antimicrobial activities against tested microorganisms including several resistant bacteria e.g., Gram-negative bacteria *P. aeruginosa* and Gram-positive bacteria MRSA and *E. faecalis*, with MIC values of 16, 8 and 32 µM each. The inhibitory concentrations against Gram-positive bacteria ranging from 4 to 32 µM seems slightly more efficient than Gram-negative ones with MICs from 8 to 32 µM. Compared with the cell wall of Gram-positive bacteria formed by peptidoglycan macromolecule constitutions, that of Gram-negative bacteria consist of a lipid and lipopolysaccharides (LPSs) in outer membrane as well as a single peptidoglycan layer. This distinction of bacterial envelope structure could be the predominant reason for different MICs of Der-PS4 [24,25]. Considering the dermaseptins published before, like Dermaseptin-PH, Der-PS4 showed stronger antimicrobial activities than Dermaseptin-PH did. The consensus motif (-GKAAGKAA-) of Der-PS4 forming higher helicity than that (-GKAA-) of Dermaseptin-PH could be one of the reasons. Moreover, the differences between the positively charged features of these two peptides might also explain the stronger antimicrobial effect of Der-PS4. Hence, favourable helicity and high cationic charge result in the noticeable antimicrobial efficiency of Der-PS4.

The biofilm inhibitory function of Der-PS4 correspond to the results of bacteria inhibitory assay on the whole. The biofilm inhibitory concentrations against *E. faecalis* and *P. aeruginosa* were relatively higher with 64 and 32 μM, respectively. This result might be due to the intrinsic resistance of these two microorganisms [26,27]. Comparing the MBEC results of the five tested pathogens, it was worthy of note that the biofilm of *P. aeruginosa* was most difficult to eradicate. It is well known that *P. aeruginosa* isolates possess a strong inherent resistance owing to the low permeability of outer membrane and notable adaptive mechanisms since biofilm forming [28,29]. These evidences may explain the phenomenon that *P. aeruginosa* exhibited high resistance against Der-PS4 after the biofilm formed with the eradication concentration at 256 μM. By a comparison of the results of MBEC and MBC, it was discovered that the MBEC values were two to eight times higher than MBC values. It is probably because the formation of biofilm could protect pathogens from external stimulations by gathering the microorganisms from planktonic organisms to sessile communities [30]. Nowadays, the mainstream of non-microbicidal methods include reducing attachment, inhibiting the formation of biofilm to enhancing permeabilized ability of antimicrobial agents or preventing the maturation of biofilm [31,32,33,34]. All these strategies focus on biofilm, which give a hint for the importance of biofilm for the survival of pathogenic bacteria. Taking this into consideration, the eradication of biofilm to prevent the formation of microbial communities provide new insights for the discovery of new antimicrobial agents.

Der-PS4 permeabilized the cell membrane of tested pathogens in two hours at low concentrations less than their MIC values. With the increase of concentration, the penetrative ability of Der-PS4 improved rapidly. It is well known that the Val-Gly pairing in dermaseptins divide the peptide into two segments forming a helix-hinge-helix structure, which can facilitate the hydrophobic residues on both extremities to insert into membrane and induce permeabilization [35,36]. While, a novel dermaseptin, named Dermaseptin-PH, discovered by Huang and co-workers, was a typical one without Val-Gly segment. Compared with Dermaseptin-PH, Der-PS4 exhibited stronger antimicrobial and penetrative activities [15]. Hence, perhaps the Val-Gly pairs within Der-PS4 was the reason why Der-PS4 could permeabilize partial cell membrane of MRSA, *E. faecalis* and *E. coli* below the MICs. In the dynamic-course figures, the penetrative procedure into the membrane could be observed clearly. For bacterium *S. aureus*, although Der-PS4 could reach full permeabilization under both 8 and 16 µM, a relative quicker membrane-disruption occurred in fifteen minutes only at the concentration of 16 μM. A similar phenomenon could also be observed in *E. coli* and *C. albicans*, which implied that the adsorption and disruption process between Der-PS4 and bacteria outer membrane took place quickly under high concentrations of Der-PS4. Galanth and colleagues presented that once the accumulation on membrane reached a threshold, the complex of positive charged peptides and anionic lipids could form a positive curvature of the bilayer and the permeation/disruption happened under the carpet mechanism [31]. These theories might explain the permeabilized results of Der-PS4, which induced the rapid disruption of the bacteria cell membrane under a relatively high concentration, indicating the accomplishment of a threshold concentration.

What’s more, Der-PS4 exhibited antiproliferative activity against five tested cancer cell lines and showed slight cytotoxicity on normal human cells. Although quite a number of dermaseptins display extensive cytotoxicity against tumour cells, it is still unclear what is the cellular selectivity or mechanism of action. The dominant perception is that the electrostatic interaction between cationic antimicrobial peptides and the anionic cancer cell membrane is the main reason [37]. The negative charged molecules located on the cancer cell membrane, such as phosphatidylserine (PS), *O*-glycoslated mucins, and glycosaminoglycans, play important roles for the interaction between the membrane and antitumour peptides [38,39,40,41]. After accumulation on the tumour cell membrane by electrostatic interaction, the peptides penetrate into the membrane and a membrane-disruption mechanism is induced followed by cell lysis. These evidences might reveal the electrostatic incorporation mechanisms of Der-PS4, which taken +3 net positive charge except the C-terminal amidation. However, the lactate dehydrogenase (LDH) release assay tested on human cancer cell lines exhibited an interesting result that Der-PS4 displayed weak potency in disrupting cancer cell membranes although high concentrations of Der-PS4 displayed strong antiproliferative activities in MTT assay, which suggested that the reduction of cell viability caused by Der-PS4 may not rely on membrane disruption but on other mechanisms. Dermaseptin-PS1, discovered by Long and colleagues, inhibited cell growth under concentrations ranging from 10^−6^ to 10^−4^ M. Whereas, only 10^−5^ and 10^−4^ M of Dermaseptin-PS1 could induce LDH release and exhibited antiproliferative activities via the induction of intrinsic apoptosis signaling merely at 10^−6^ M [42]. Der-PS4 could reduce cell viability ranging from 10^−8^ to 10^−4^ M while induced LDH release at 10^−5^ and 10^−4^ M. Hence, it is reasonable to deduce that the anticancer mechanisms of Der-PS4 are the joint effect of both electrostatic interaction and other antineoplastic pathways depending on different concentrations.

## 4. Materials and Methods

### 4.1. Acquisition of Phyllomedusa sauvagii Skin Secretions

The waxy monkey tree frogs, *Phyllomedusa sauvagii*, were fed with multivitamin-loaded crickets three times per week in the new breeding environment for three months or more before the experiment. All frogs were housed in separate under 12 h/12 h day/night cycles. The secretions were obtained after the gentle transdermal electrical stimulation, and then washed by deionised water and collected in a glass beaker. Afterwards, the specimens were quick-frozen in liquid nitrogen and then lyophilised. The products were kept at −20 °C prior to the next step [43]. The procedure was carried out based on the guidelines in the UK Animal (Scientific Procedures) Act 1986, project license PPL 2694, issued by the Department of Health, Social Services and Public Safety, Northern Ireland. The operational process was supervised by the IACUC of Queen’s University Belfast, and authorized on 1 March 2011.

### 4.2. Molecular Cloning and Structural Characterisation of Der-PS4 from a Skin Secretion-Derived cDNA Library of Phyllomedusa sauvagii

Five milligrams of lyophilised skin secretions were dissolved in 1ml lysis/binding buffer (Dynal Biotech, Merseyside, UK) followed by isolation using a magnetized Dynabeads^®^ mRNA Direct^TM^ Kit (Dynal Biotech, Merseyside, UK) based on A-T pairing. The isolated mRNA acted as a template and the first strand of cDNA library was constructed using a SMART-RACE Kit (Clontech, Palo Alto, CA, USA). Then the RACE-PCR was performed by using a nested universal primer (NUP) supplied from the kit as antisense primer and the degenerate sense primer (S1; 5′-ACTTTCYGAWTTRYAAGMCCAAABATG-3′) (Y = C/T; W = A/T; R = A/G’ M = A/C; B = T/C/G) which was designed based on nucleotide sequences of the highly conserved signal peptide region of dermaseptin peptides published previously from *Phyllomedusa sauvagii*. The PCR products were gel-electrophoresis analyzed and purified by using a Concert^TM^ Rapid PCR Purification System (Life Technologies, Warrington, UK). Then the purified products were cloned using a pGEM-T Easy Vector System (Promega Corporation, Southampton, UK) and gel-electrophoresis analyzed followed by purification using the Concert^TM^ Rapid PCR purification System. Finally, the sequencing reaction was carried out using a BigDye Terminator Sequencing Kit (Applied Biosystems, Foster City, CA, USA). An ABI 3100 automated capillary sequencer (Applied Biosystems, Foster City, CA, USA) was engaged to analyse the nucleotide sequence of selected cloned products.

### 4.3. Identification and Structural Chracterisation of the Predicted Der-PS4 from the Skin Secretion of Phyllomedusa sauvagii

Five milligrams of lyophilized skin secretions were dissolved in 1ml of water/trifluoracetic acid (TFA) (99.95/0.05, *v*/*v*) and then centrifuged at 5000 rpm for 20 min. The supernatant was collected and injected into an RP-HPLC system (Waters, Miford, MA, USA) loaded with a column (Jupiter C-18, 5 µM, 4.6 mm × 250 mm, Phenomenex, Macclesfield, Cheshire, UK). Samples were eluted with a linear gradient mobile phase from water/trifluoracetic acid (TFA) (99.95/0.05, *v*/*v*) to acetonitrile/water/TFA (80.00/19.95/0.05, *v*/*v*) at a flow rate of 1ml/min for 240 min. The absorbance was detected at 214 and 280 nm wavelength. The fractions were collected every minute and then analysed by MALDI-TOF (matrix assisted laser dissociation ionized-time of flight) (Voyager DE, Perspective Biosystem, Foster City, CA, USA) with CHCA (α-cyano-4-hydroxycinnamic acid) as matrix. Then, fractions containing molecular mass coincident with the peptide identified from “shot-gun” cloning result were injected into an LCQ-Fleet electrospray ion-trap mass spectrometer (Thermo Fisher Scientific, San Francisco, CA, USA) to confirm the amino acid sequences of predicted mature peptide using MS/MS fragmentation sequencing technique.

### 4.4. Solid-Phase Synthesis of Der-PS4

Dermaseptin-PS4 was synthesized using Fmoc (9-fluorenylmethyloxycarbonyl) solid-phase chemistry synthesis technique using a Tribute^®^ PS4 Peptide Synthesizer (Protein Technologies, Tucson, AZ, USA). HBTU (2-(1H-benzotriazol-1-yl)-1,1,3,3-tetramethyluronium hexafluorophosphate) acted as an activator and peptides were deprotected from Fmoc protection groups using dimethylformamide/piperidine (80/20, *v/v*). The cleavage process was carried out in a cocktail consisted of TFA/thioanisole/H_2_O/1,2-ethanedithiol (94/2/2/2, *v/v/v/v*) at room temperature for maximum 120 min. Following that, peptides were washed using diethyl ether and dissolved in TFA/water (99.95/0.05, *v/v*) before lyophilisation. The lyophilised products were purified using RP-HPLC and the purity result was identified by mass spectrometry. The purified products were stored at −20 °C before bioactive assessment.

### 4.5. Secondary Structural Analysis of Der-PS4 through Circular Dichroism (CD) Spectroscopy

The second structure of Der-PS4 was analyzed by circular dichroism spectroscopy technique using a JASCO J-815 CD spectrometer (Jasco, Essex, UK), as described previously [44]. Basically, synthesized Der-PS4 was prepared at 50 μM in 10 mM ammonium acetate, TFE/10mM ammonium acetate (50/50, v/v) and large unilamellar vesicles (LUVs) solutions for conformational study. Stock solutions of DOPG, DOPC and DOPE (Avanti, Alabaster, AL, USA) were prepared at 1mg/mL in chloroform. Desired proportions were mixed and evaporated using vacuum rotary evaporator (IKA, Staufen, DE) and the residual solvent was removed in a vacuum chamber. The dried lipid film was collected and dissolved in MiliQ water at PH 6.5. The LUVs were obtained after five cycles of freeze-thawing in liquid nitrogen followed by extrusion through two stacks of 0.2 μM polycarbonate filters (Millipore Corp., Bedford, MA, USA) using a Liposofast low pressure homogenizer (Avestin, Ottawa, CA). The lipid concentration of LUVs subjected to CD analysis was 0.5mM. Parameters were set as 1 nm bandwidth and 0.5 nm data pitch and the data was recorded at a wavelength ranging from 190 nm to 260 nm with a 200 nm/min scan speed. Secondary structure results were estimated by BESTSEL CD spectrum analysis tool [45]. The structural parameters of Der-PS4 including physicochemical properties, polar residues, nonpolar residues and helical wheel projections were acquired by analyzing on Heliquest (http://heliquest.ipmc.cnrs.fr/).

### 4.6. Minimum Inhibitory Concentration (MIC) and Minimum Bactericidal Concentration (MBC) Assays

Gram-positive bacteria *Staphylococcus aureus* (NCTC 10788), methicillin-resistant *Staphylococcus aureus* (MRSA) (NCTC 12493) and *Enterococcus faecalis* (NCTC 12697); Gram-negative bacteria *Escherichia coli* (NCTC 10418) and *Pseudomonas aeruginosa* (ATCC 27853); fungus *Candida albicans* (NCYC 1467) was inoculated in Mueller-Hinton broth (MHB) (Sigma-Aldrich, St. Louis, MO, USA) for sixteen to eighteen hours. Then 500 µL of initial growth cultures were transferred into 20 mL MHB and cultured in a shaking incubator at 37 °C until the microorganisms reaching logarithmic growth phases by testing the optical density (OD) at 550 nm wavelength. After that, the subcultured microorganisms were diluted by MHB until 1 × 10^6^ colony forming units (cfu)/mL for bacteria and 1 × 10^5^ cfu/mL for fungus obtained. Then the microorganisms were cultured with Der-PS4 in a range of final concentrations from 1 µM to 512 µM in a 96-well culture plate. The plate was incubated at 37 °C for sixteen to eighteen hours in a humid atmosphere. The growth curve of each microorganism was determined by means of measuring absorbance values at 550 nm wavelength in a Synergy HT plate reader (Biotech, Minneapolis, MN, USA). The MIC value was defined as the lowest concentration of Der-PS4 at which no obvious growth of each microorganism was observed. For the MBC assay, transfer 20 µL microbial suspension from MIC to each higher concentration onto a Mueller-Hinton agar (MHA) (Sigma-Aldrich, St. Louis, MO, USA) plate. After eighteen hours incubation at 37 °C, the MBC value was defined as the lowest concentration of Der-PS4 at which no obvious colonies were observed.

### 4.7. Minimum Biofilm Inhibitory Concentration (MBIC) and Minimum Biofilm Eradication Concentration (MBEC) Assays

Gram-positive bacteria *S. aureus* (NCTC 10788), MRSA (NCTC 12493) and *E. faecalis* (NCTC 12697) were inoculated in tryptic soy broth (TSB) (Sigma-Aldrich, St. Louis, MO, USA) for sixteen to eighteen hours at 37 °C in a shaking incubator at different revolutions per minute (rpm) according to different bacteria. Gram-negative bacteria *E. coli* (NCTC 10418) and *P. aeruginosa* (ATCC 27853) were inoculated in Luria-Bertani (LB) broth (Sigma-Aldrich, St. Louis, MO, USA) for sixteen to eighteen hours at 37 °C in a shaking incubator at different revolutions per minute (rpm) according to different bacteria. Then 500 µL of initial growth cultures were transferred into 20 mL relevant broth and cultured in a shaking incubator at 37 °C until the microorganisms reaching logarithmic growth phases by testing the OD at 550 nm. After that, the subcultured microorganisms were diluted by relevant broth until 1 × 10^6^ cfu/mL density obtained and ready for subsequent assays. For the MBIC assay, each microorganism was cultured with Der-PS4 in a range of final concentrations from 1 µM to 512 µM in a 96-well flat-bottom culture plate. Plate was incubated at 37 °C in a humid atmosphere at relevant rpm. Sixteen to eighteen hours later, the plate was washed twice by phosphate buffer saline (PBS) (Sigma-Aldrich, St. Louis, MO, USA) and fixed with methanol for 10 min. For the MBEC assay, 100 µL of each microorganism was transferred to a 96-well flat-bottom culture plate and incubated at 37 °C in a humid atmosphere at relevant rpm. Sixteen to eighteen hours later, the plate was washed twice by PBS and re-cultured with 100 µL Der-PS4 dissolved in relevant culture medium in a range of concentrations from 1 µM to 512 µM. Sixteen to eighteen hours after exposure, plate was washed twice again with PBS and fixed with methanol for 10 min. Subsequently, 150 µL 0.1% (*m*/*v*) crystal violet solution was used to stain the biofilm of both MBIC and MBEC plates. After 30 min, plates were washed by PBS until no obvious crystal violet solution was observed. After evaporation of the stained plates, 100 µL 30% (*v*/*v*) acetic acid were used to dissolve the stains and measured at 595 nm wavelength using a Synergy HT plate reader (Biotech, Minneapolis, MN, USA). The MBIC value was defined as the minimum concentration which inhibited the formation of biofilm over 90% compared to the negative control. The MBEC value was defined as the minimum concentration which eradicated the formed biofilm over 90% compared to the negative control [46].

### 4.8. Haemollysis Assay

Two millilitres defibrinated horse blood (TCS Biosciences Ltd., Botolph Clayton, UK) was centrifuged at 1000× *g* for five minutes. Then, the supernatant was discarded and washed by PBS until the supernatant was clear. The final supernatant was quitted and the blood cells were resuspended by PBS until a concentration of 4% (*v/v*) obtained. Then, the resuspended cells were incubated with Der-PS4 in a range of final concentrations from 1 µM to 512 µM at 37 °C for 2 h. After the exposure, the mixtures were centrifuged at 1000× *g* for five minutes to collect supernatant and placed into a 96-well plate. The absorbance was detected at 570 nm wave length by a Synergy HT plate reader (Biotech, Minneapolis, MN, USA). Blood cells treated with 1% (*v/v*) Triton-*X* 100 (Sigma-Aldrich, St. Louis, MO, USA) were used as positive control.

### 4.9. Bacteria Cell Membrane Permeability Assay

Each microorganism was inoculated in TSB (Sigma-Aldrich, St. Louis, MO, USA) for sixteen to eighteen hours at 37 °C in a shaking incubator at different revolutions per minute (rpm) according to different bacteria. Then 200 µL of initial growth cultures were transferred into 25 mL TSB and cultured in a shaking incubator at 37 °C. 2.5 h later, the cultures were centrifuged at 1000× *g* at 4 °C for 10 min to collect the bacteria and then washed twice by 30 mL 5% *(v/v)* TSB in 0.85% *(m/v)* sodium chloride (NaCl) solution. Then, the washed bacteria were resuspended by 5% *(v/v)* TSB in 0.85% *(m/v)* NaCl solution until a 1 × 10^8^ cfu/mL density obtained by testing the OD at 590 nm wavelength. The microorganisms were incubated with Der-PS4 in a range of concentrations based on MICs in a black 96-well flat bottom plate at 37 °C for two hours. For the positive control, the microorganisms were incubated with 70% (*v/v*) isopropanol at room temperature for 1 h and then centrifuged at 13,000 × *g* at 4 °C for 5 min. The supernatant was discarded and the precipitate was washed twice by 5% *(v/v)* TSB in 0.85% *(m/v)* NaCl solution and finally resuspended by 5% *(v/v)* TSB in 0.85% *(m/v)* NaCl solution. All groups were stained by SYTOX^TM^ green nucleic acid stain (Life technologies, Carlsbad, CA) to a final concentration of 5 µM and incubated in dark at 37 °C for 5 min. The fluorescent intensity was detected by a Synergy HT plate reader (Biotech, Minneapolis, MN, USA) with the excitation of 485 nm and emission of 528 nm.

For the time-curve bacteria cell membrane permeability assay, the microorganisms were processed as described above until a 1 × 10^8^ cfu/mL density obtained. After that, the positive control was treated with 70% (*v/v*) isopropanol at room temperature for 1 h first, then washed twice and resuspended by 5% *(v/v)* TSB in 0.85% *(m/v)* NaCl solution. All groups were incubated with SYTOX^TM^ green stain (5 µM) for 15 min and subsequently were treated by Der-PS4 with final concentration of 4, 8 and 16 µM respectively based on the results of last step. The fluorescent intensity was detected every 15 min for 120 min.

### 4.10. Scanning Electron Microscope (SEM) Characterisation

Bacterial cells were collected by centrifugation at 1000 ×g for 10 min, washed thrice with 0.1 M PBS (pH = 7.4), and resuspended to an OD at 600 nm wavelength of 0.2 with PBS. Cells were incubated with peptides at 37 °C for 15 min at their 1 × MICs. The control was run without peptides. After incubation, the cells were collected, washed with PBS three times and fixed with 2.5% (*m*/*v*) glutaraldehyde at 4 °C overnight. Then, cells were washed twice with PBS. The cells were dehydrated for 15 min in a graded ethanol series, followed by 15 min in 100% ethanol, a mixture (1/1, *v*/*v*) of 100% ethanol and tert-butanol, and absolute tert-butanol. Finally, the specimens were dehydrated in a critical point dryer with liquid CO_2_, coated with gold–palladium, and then observed using a Hitachi SU8010 scanning electron microscope (Hitachi, Tokyo, Japan).

### 4.11. MTT Cell Viability Assay

U251MG (ECACC-09063001), MCF-7 (ATCC-HTB-22) and MDA-MB-435s (ATCC-HTB-129) were cultured in Dulbecco’s Modified Eagle Medium (DMEM) (Life technologies, Carlsbad, CA) supplemented with 10% (*v/v*) fetal bovine serum (FBS) (Sigma-Aldrich, St. Louis, MO, USA) and 1% (*v/v*) penicillin-streptomycin (Invitrogen, Paisley, UK). H157 (ATCC-CRL-5802) and PC-3 (ATCC-CRL-1435) were cultured in Roswell Park Memorial Institute (RPMI)-1640 (Life technologies, Carlsbad, CA) supplemented with 10% (*v/v*) FBS and 1% (*v/v*) penicillin-streptomycin. HMEC-1 (ATCC-CRL-3243) was cultured in MCDB131 (Life technologies, Carlsbad, CA) supplemented with 10% (*v/v*) FBS, 1% (*v/v*) penicillin-streptomycin, 10 ng/mL epidermal growth factor (EGF), 10 mM _L_-glutamine and 1 μg/mL hydrocortisone. Cells were grown in a 96-well flat-bottom culture plate in a density of 3000 cells per well. After 24 h, cells were treated with relevant culture medium without FBS and cultured for 12 h. Then, cells were treated with Der-PS4 in a range of concentrations from 10^−9^ to 10^−4^ M for 24 h. Subsequently, MTT (3-(4,5-Dimethylthiazol-2-yl)-2,5-Diphehyltetrazolium Bromide) was added to each well to a final concentration of 0.5 mg/mL and incubated at 37 °C. Six hours after exposure, mixtures in each well were discarded and 100 μL DMSO was added. The absorbance was detected at 570 nm wavelength using a Synergy HT plate reader (Biotech, Minneapolis, MN, USA).

### 4.12. Lactate Dehydrogenase (LDH) Cytotoxicity Assay

Cells were cultured as described in Section 4.10. Cells were grown in a 96-well flat-bottom culture plate in density of 3000 cells per well and treated with 10^−9^ to 10^−4^ M of Der-PS4 for 24 h. Cells treated with lysis buffer supplied from Pierce LDH Cytotoxicity Assay Kit (Thermo Fisher Scientific, Loughborough, UK) at 37 °C for 45 min were acted as maximum LDH activity controls. Then 50 μL cultured medium were mixed with 50 μL reaction mixture at room temperature for 30 min keeping in a dark place. Subsequently, 50 μL stop solution was added by gentle pipetting. The absorbance was measured at 490 nm and 680 nm wavelength respectively using a Synergy HT plate reader (Biotech, Minneapolis, MN, USA).

## Figures and Tables

**Table 1:** Structural parameters of Der-PS4.

Der-PS4: _1_ALWKTLLKHVGKAAGKAALNAVTDMVNQ_28_			
**Physico-Chemical Properties**	**Hydrophobicity (H)** **0.368**	**Hydrophobic Moment <** **μH** **>** **0.226**	**Net Charge** 3
**Polar residues** **+ GLY**	**Polar residues + GLY (n/%)** 13/46.43	**Uncharged residues + GLY** GLN 1, HIS 1, THR 2, ASN 2, GLY 2	**Charged residues** LYS 4, ASP 1
**Nonpolar** **residues**	**Nonpolar residues (n/%)** 15/53.57	**Aromatic residues** TRP 1	**Special residues** CYS 0, PRO 0
**Hydrophobic face:** W A A L M A			

**Table 2:** MICs and MBCs of Der-PS4, ampicillin and norfloxacin.

Drugs	MIC/MBC (µM)					
	*S. aureus*	MRSA	*E. faecalis*	*E. coli*	*P. aeruginosa*	*C. albicans*
**Der-PS4**	4/8	8/16	32/32	8/16	16/32	4/16
**Melittin**	2/2	2/4	2/2	2/4	32/32	4/4
**Ampicillin**	0.3/0.3	-	4.8/4.8	36.6/36.6	-	146/-
**Norfloxacin**	1.3/2.5	2.5/5.2	5.2/5.2	0.6/0.6	2.5/5.2	1.3/2.5

**Table 3:** The minimum biofilm inhibitory concentrations (MBICs) and minimum biofilm eradication concentrations (MBEC) of Der-PS4.

	*S. aureus*	MRSA	*E. faecalis*	*E. coli*	*P. aeruginosa*
**MBIC (μM)**	4	8	64	8	32
**MBEC (μM)**	64	64	128	32	256

## Abbreviations

The following abbreviations were used in this manuscript:

RP-HPLC	reverse-phase high-performance liquid chromatography
SPPS	solid-phase peptide synthesis
CD	circular dichroism
TFE	9-fluorenylmethyloxycarbonyl
SEM	scanning electron microscopy
MTT	3-(4,5-dimethyl-2-thiazolyl)-2,5-diphenyl-2-H-tetrazolium bromide
LDH	lactate dehydrogenase
RACE-PCR	rapid amplification of cDNA ends-polymerase chain reaction
NUP	nested universal primer
TFA	trifluoracetic acid
MALDI-TOF	matrix-assisted laser desorption/ionization time-of-flight
CHCA	*α*-cyano-4-hydroxycinnamic acid
Fmoc	9-fluorenylmethyloxycarbonyl
HBTU	2-(1H-benzotriazol-1-yl)-1,1,3,3-tetramethyluronium hexafluorophosphate
OD	optical density
